# Jaccard distance based weighted sparse representation for coarse-to-fine plant species recognition

**DOI:** 10.1371/journal.pone.0178317

**Published:** 2017-06-07

**Authors:** Shanwen Zhang, Xiaowei Wu, Zhuhong You

**Affiliations:** 1 Department of Information Engineering, Xijing University, Xi’an, China; 2 Department of Statistics, Virginia Tech, Blacksburg, Virginia, United States of America; Soochow University, CHINA

## Abstract

Leaf based plant species recognition plays an important role in ecological protection, however its application to large and modern leaf databases has been a long-standing obstacle due to the computational cost and feasibility. Recognizing such limitations, we propose a Jaccard distance based sparse representation (JDSR) method which adopts a two-stage, coarse to fine strategy for plant species recognition. In the first stage, we use the Jaccard distance between the test sample and each training sample to coarsely determine the candidate classes of the test sample. The second stage includes a Jaccard distance based weighted sparse representation based classification(WSRC), which aims to approximately represent the test sample in the training space, and classify it by the approximation residuals. Since the training model of our JDSR method involves much fewer but more informative representatives, this method is expected to overcome the limitation of high computational and memory costs in traditional sparse representation based classification. Comparative experimental results on a public leaf image database demonstrate that the proposed method outperforms other existing feature extraction and SRC based plant recognition methods in terms of both accuracy and computational speed.

## Introduction

With the continuous development of the digital image processing, pattern recognition and computer software and hardware technologies, more and more studies have been presented on plant species classification and recognition [1,2]. Du et al. [3] proposed a leaf classification method called Move Median Centres (MMC) hyper sphere classifier. The experimental results show that the method can save storage space and reduce the classification time. Hu et al. [4] proposed a contour-based leaf shape descriptor to capture the leaf shape characteristics. The extracted characteristics are invariant to the translation, rotation, scaling and bilateral symmetry. Kadir et.al [5] proposed a plant species recognition method by extracting the various features such as texture, vein, shape and color of the leaves. Kumar et al. [6] proposed a medicinal plant recognition system by the edge features of plant leaves. The method is limited to detect only the mature leaves since the tender leaves changes slightly when it becomes mature. Zhang et al. [7] proposed a supervised locality projection analysis (SLPA) method for plant classification by making use of the label propagation. Du et al. [8] developed a leaf recognition method by extracting the features from the image sets rather than from the individual image. The leaf shape information is extracted by pyramid of histograms of orientation gradients (PHOG) descriptor, and then each image set is characterized from the image set, and the plant species are recognized by the minimum distance between manifolds.

**Fig 1:**
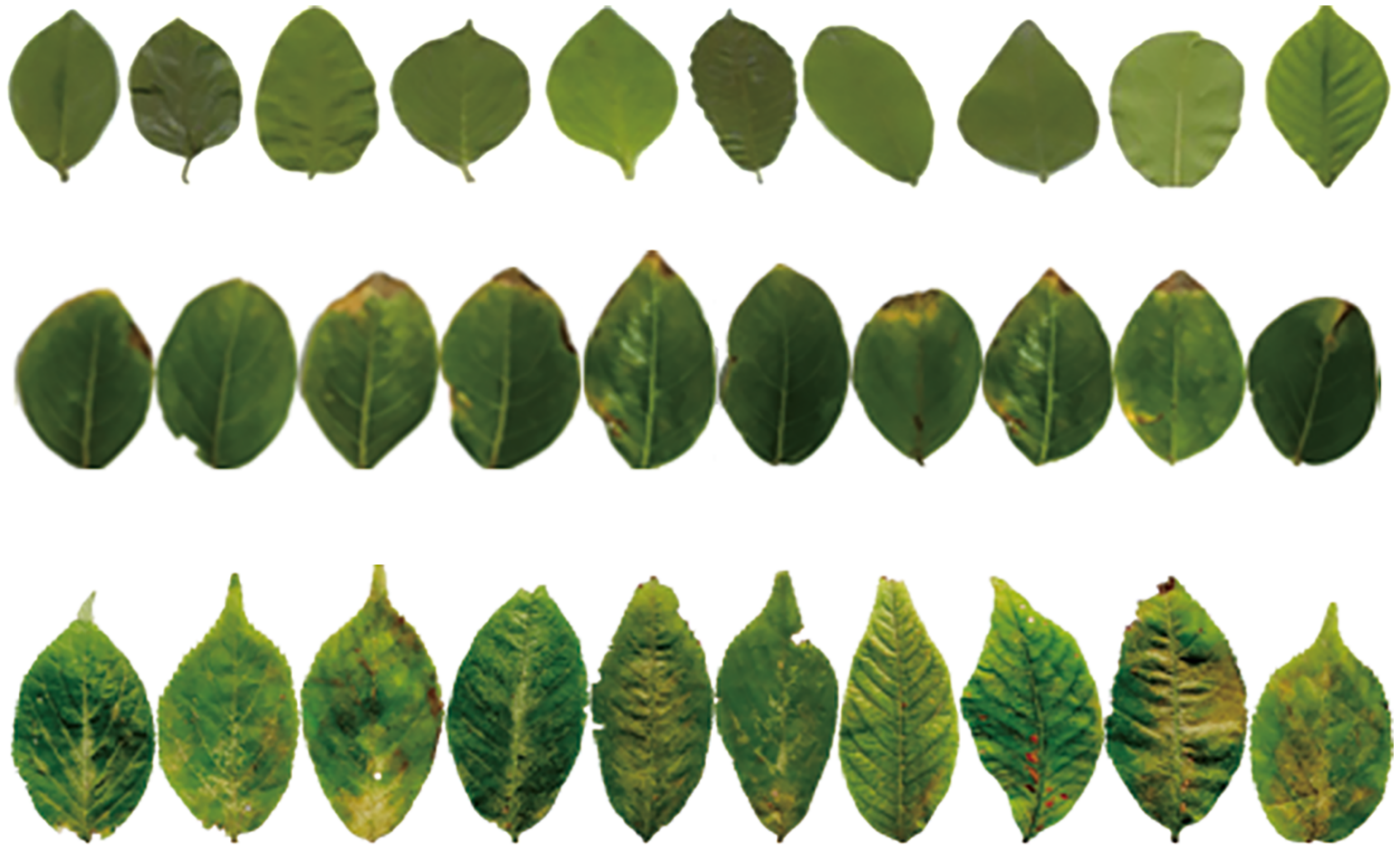
Some plant leaves.

From the above papers, it is known that the key issue of the plant species recognition lies in whether the extracted and selected features are stable and have good ability to discriminate the plant leaves. But in fact, many classical plant recognition methods have not been applied to the plant species recognition system because of a lot of factors, such as, the leaf images are much complex, various and irregular, and the color, shape and texture of the leaf are various with the seasons and lighting level, specially the leaves collected from the same tree are different from each other. Although a number of kinds of the leaf features can be extracted for plant species recognition, by now, it is not clear which kinds of features are optimal and necessary. In recent years, deep learning has led to a series of breakthroughs in many various research areas, such as computer vision, image recognition, speech recognition, target classification and detection, and has been successfully applied to plant recognition [9,10]. Deep learning can automatically learn the intrinsic features from the original image, which can overcome the difficulty of feature extraction in the traditional recognition methods. However, deep learning based methods needs to build a special platform with hardware and software framework and deep learning based methods usually take longer time to do the recognition task. Sparse representation (SR) based classification (SRC) has been widely and success fully applied to face recognition and image annotation, and so on [11–13]. Dictionary learning plays a crucial role in sparse representation based image classification. Li et al. [14] proposed a novel approach to learn a discriminative dictionary with low-rank regularization on the dictionary. Li et al. [15] proposed a cross-view projective dictionary learning (CPDL) approach to capture the intrinsic relationships of different representation coefficients in various settings. In SRC, in theory, the test sample can be well represented by only the training samples of the same class, but in the large leaf image database, a number of the different kinds of species leaves may be very similar (as shown in Fig 1A) and a number of the same kind of species leaves may be very different(as shown in Fig 1B), moreover there are a lot of unusual, irregular, crippled, corrupted and even disease leaves (as shown in Fig 1C), which will cause the sparse coefficients not to be sparse [16,17].

As for the leaf based plant species recognition on the large-scale dataset, in designing a classification algorithm, it is reasonable to assume that if a given leaf image is similar to the test leaf image, it has great effect on the classification decision of the test leaf image; conversely, if a leaf image is very dissimilar to the test leaf image, it has little effect or even has side-effect on the classification decision of the test leaf image, so the different leaf should be imposed different levels of importance or weight, and some leaves should be directly excluded from the training set. By this justification and inspired by SR based coarse-to-fine face recognition [11], a new coarse-to-fine plant species recognition method is proposed by Jaccard distance based spare representation (JDSR) method. The main highlight is that half of the training samples are directly excluded by the Jaccard coefficient and WSRC is implemented on a few training samples, instead of processing each leaf image in the same way in the whole plant species recognition process.

The main contributions are (1) the Jaccard coefficient is utilized to exclude half of the training classes that are far from the test sample. So the side-effect on the classification decision is eliminated; (2) weighted Jaccard distance based SR (JDSR) is proposed to compactly represent the test data in terms of the few candidate classes; (3) the representation result of JDSR is sparser than that of the classical SR algorithm.

The rest of the paper is organized as follows: Section 2 briefly introduces the related works, including the classical SR, the weighted SR, and the Jaccard metric. We provide details of the proposed coarse-to-fine plant species recognition method in Section 3, and present the experimental results in Section 4. Finally, conclusions and further works are summarized in Section 5.

## Related works

### Sparse representation (SR)

Mathematically, solving the SR problem involves seeking the sparsest linear combination from an over-complete dictionary. Suppose, in a classification problem, there are *n* training samples from *C* classes, denoted by column vectors *G* = [*g*_1_,*g*_2_,…,*g*_*n*_]∈*R*^*m*×*n*^, and a test sample *y*∈*R*^*m*^. For classification purpose, the test sample *y* can be represented by a linear combination of *G* as follows,

$$y=\alpha_{1}g_{1}+\alpha_{2}g_{2}+\cdots +\alpha_{n}g_{n}$$ (1)

where *α*_*i*_(*i* = 1,2,…,*n*) is the SR coefficient. In the matrix form, Eq (1) can be rewritten as

$$y=[g_{1},g_{2},\ldots ,g_{n}]{\left[\alpha_{1},\alpha_{2},\cdots ,\alpha_{n}\right]}^{T}=GA$$ (2)

where *A* = [*α*_1_,*α*_2_,…,*α*_*n*_]^*T*^. SR based classification aims to find the class that produces the most “accurate” sparse representation of the test sample. In Eq (1), *α*_*i*_*g*_*i*_ is regarded as the contribution of training sample *g*_*i*_ to representing test sample *y*. Accuracy of this sparse representation in each class can be evaluated by the deviateon *e*_*i*_(*y*) = ||*y* = *α*_*i*_*g*_*i*_||^2^, *i*∈*C*, which can also be somewhat viewed as the weighted distance between *g*_*i*_ and *y*. Smaller *e*_*i*_(*y*) means that the training samples in the *i*th class have a greater contribution to the sparsely representation of *y*. Taking into account the sparsity in the coefficients, the SRC can be obtained by solving the following mixed *l*_2_–*l*_1_ norm minimization problem [11,13],

$$J(A,\mu )=\underset{A}{\text{min}}{\left\|GA-y\right\|}_{2}^{2}+\mu {\left\|A\right\|}_{1}$$ (3)

where ${\left\|x\right\|}_{2}=\sqrt{\sum_{i}{\left|x_{i}\right|}^{2}}$ and ||*x*||_1_ = Σ_*i*_|*x*_*i*_| are the *l*_2_-norm and *l*_1_-norm, respectively, and *μ* is a scalar regularization parameter to balance the trade-off between the *l*_2_-norm minimization reconstruction error and the *l*_1_-norm of the coefficients used to reconstruct *y*.

The coefficient vector *A* can be obtained by *A* = (*G*^*T*^ *G*+*μI*)^−1^ *G*^*T*^ *y*, where *I* is an identity matrix [12].

### Weighted SR based classification (WSRC)

SRC works well in the fact applications when the test sample can be reconstructed as accurately as possible. However, the typical SRC does not consider the prior information between the test sample and the training samples. To improve the performance of SRC, some weighted SRC (WSRC) algorithms have been proposed, in which a weight is utilized to enhance the contribution of the training samples to representing the test sample. The weight value can be determined by various distance and similarity measurements, for example, the cosine similarity [18], the Euclidean distance [19] and the Gaussian kernel distance [20]. In general, WSRC solves the following weighted mixed *l*_2_−*l*_1_ norm minimization problem,

$$J(A,\mu )=\underset{A}{\text{min}}{\left\|GA-y\right\|}_{2}^{2}+\mu {\left\|WA\right\|}_{1}$$ (4)

where *W*∈*R*^*n*×*n*^ is a weighted diagonal matrix with diagonal elements denoted as *w*_1_,*w*_2_,…,*w*_*n*_. It is clear that when *w*_*i*_(*i* = 1,2,…,*n*) in WSRC are all set to 1, WSRC is indeed SRC. Both SRC and WSRC are the constrained LASSO problem [20,21].

### Jaccard distance of binary images

In image analysis, different (e.g., Euclidean, Mahalanobis, cosine, Gaussian kernel, and Jaccard) distance metrics have been used to calibrate the similarity between images [22]. Specifically, for binary images the Jaccard distance can be calculated in a simple and fast way. Given two binary images *G*_1_ and *G*_2_, the Jaccard coefficient (similarity) *J* and Jaccard distance (dissimilarity) *d*_*J*_ are defined respectively as follows,

$$J(G_{1},G_{2})=\frac{M_{11}}{M_{01}+M_{10}+M_{11}}$$ (5)

$$d_{J}(G_{1},G_{2})=1-J(G_{1},G_{2})=\frac{M_{01}+M_{10}}{M_{01}+M_{10}+M_{11}}$$ (6)

where *M*_11_ is the total number of pixels on which both *G*_1_ and *G*_2_takevalue 1,and similarly, *M*_01_ is the total number of pixels on which *G*_1_ takes value 0 but *G*_2_ takes value1, *M*_10_ is the total number of pixels on which*G*_1_takes value 1 and *G*_2_ takes value 0. From Eqs (5) and (6), the Jaccard metric eliminates the matching pixels that share the value of 0, which makes it very adequate for evaluating the similarity between leaf texture images, because in these images most of the pixels are 0's. For example, suppose two binary vectors *V*_1_ = [1 0 0 1 0 0 1 1 0 0] and *V*_2_ = [0 0 1 1 0 1 0 1 1 0], then *M*_11_ = 2,*M*_01_ = 3,*M*_10_ = 2. From Eqs (5) and (6), the Jaccard coefficient and Jaccard distance are computed as 2/7 = 0.2857 and 5/7 = 0.7143, respectively. In fact, the MATLAB function pdist(X, 'jaccard') can be used to compute the Jaccard similarity.

## Coarse-to-fine plant species recognition

The texture and shape are two kinds of the most important features for describing the leaves in leaf based species automatic recognition. The two kinds of features can be represented by the binary images of leaf, which are used as distinctive features for species recognition [3,7]. We convert all of the original color leaf images into grayscale images, and then extract the binary edge orientation images by the Canny edge detection algorithm, which can be implement by the function edge (I, 'Canny', threshold) in MATLAB 7.0. For leaf classification on the large database, the accurate reconstruction by SR is nearly impossible. For this purpose, a coarse-to-fine leaf based plant recognition method is proposed in detail in this section. In the method, the training samples are coarsely classified by Jaccard coefficient, and then are finely recognized by Jaccard distance based SR. The binary edge orientation images are used in the coarse-to-fine plant recognition.

### Coarse recognition by Jaccard coefficient

In plant species recognition, the samples are usually binary leaf texture images, which can be easily extracted from the original color leaf images by using the Canny edge detection algorithm [23]. The key problem in the recognition is to calculate the distance between the binary leaf orientation images. From Eqs (5) and (6), the Jaccard distance serves as a suitable distance measure for this purpose.

As an example, let us consider 11 training leaf images from 5 different species and a test leaf image from the first species, as shown in Table 1. We first convert the color leaf images into grayscale images, and then extract the binary orientation images by the Canny edge detection algorithm, which can be implemented by the function edge(I,'Canny',threshold) in MATLAB 7.0. By Eqs (5) and (6), we calculate the Jaccard coefficient and Jaccard distance between the test orientation image and each of the 11 training orientation images, respectively.

**Table 1:** The Jaccard coefficients and Jaccard distances between the test image and 11 training leaf images.

Species No.	No. 1	No. 2	No.3,4, 5
Jaccard coefficient	0.681 0.732 0.764 0.741, Average is 0.7295	0.618 0.662 0.597 0.462, Average is 0.5847	0.181 0.169 0.118
Jaccard distance	0.319 0.268 0.236 0.259, Average is 0.2705	0.482 0.438 0.403 0.538, Average is 0.4153	0.819 0.731 0.882

From Table 1, it is found that the Jaccard coefficients of Species 1 are the largest, so the test leaf image can be basically classified into Species 1. But it also probably belongs to Species 2, as its Jaccard coefficients with Species 2 are also relatively larger. It is unlikely that the test leaf image belongs to the Species 3, 4, and 5, because their Jaccard coefficients are the three smallest according to Table 1. Therefore, the Jaccard coefficient can be used to coarsely exclude training samples which are dissimilar to the test sample and determine the candidate class labels of the test sample. To reduce bias from unusual or irregular leaf images, the average Jaccard coefficient of each training class is utilized to classify the test sample, which is defined as follows,

$$J(y,X_{i})=\frac{1}{n_{i}}\sum_{x_{j}\in X_{i}}^{}J(y,x_{j})$$ (7)

where *n*_*i*_ is the number of training samples in the *i*th class *X*_*i*_. If the average Jaccard coefficient *J*(*y*,*X*_*i*_) is small, it indicates that the training samples of the *i*th class (*i* = 1,2,…,*C*) have smaller contribution or even have side-effect in the leaf based plant species recognition, so all of the samples of *X*_*i*_ should be excluded from the training set.

In coarse recognition stage, we first convert each color leaf image into grayscale image and extract its binary orientation image, calculate the average Jaccard coefficient of each class, and then simply select *S* classes with top ranked (from large to small) average Jaccard coefficients as the candidate classes of the test sample, while directly exclude the training samples from the other *C-S* classes. In practice we set *S<C*/2. Since more than half of the training classes are excluded, the subsequent fine recognition stage becomes simpler and clearer.

### Fine recognition by Jaccard distance based SR

Although existing WSRC algorithms have been successfully applied to face recognition [18–20], their direct application to plant leaf species recognition is often problematic, because leaf images are more complex and various than human face images. As a simple distance metric, the Jaccard distance can be used to quickly measure the similarity or dissimilarity between two binary images, which provides a natural weighting strategy for the training leaf orientation images based on WSRC.

Following the coarse recognition stage, let us assume that *S* candidate classes have been reserved, simply denoted as *X*_1_,*X*_2_,…,*X*_*S*_, and the number of training samples in the *i*th class is denoted as *n*_*i*_ (*i* = 1,2,…,*S*). In practice, the test sample and each training sample of *X*_1_,*X*_2_,…,*X*_*S*_ are transformed into one-dimensional column vectors, denoted as *y* and *x*_1_,*x*_2_,…,*x*_*m*_, respectively, where *y*∈*R*^*D*^,*x*_*i*_∈*R*^*D*^, *D* is the dimension of the column vector representation, *m* = *n*_1_+*n*_2_+…+*n*_*S*_ is the total number of reserved training samples. The dictionary of sparse representation is constructed by concatenating *x*_1_,*x*_2_,…,*x*_*m*_ as a matrix *G*∈*R*^*D*×*m*^, where each column of *G* represents a candidate training image, known as an atom. Based on the Jaccard distance, we propose a new WSRC to solve the optimization problem in Eq (4),

$$J(A,\mu )=\underset{A}{\text{min}}{\left\|GA-y\right\|}_{2}^{2}+\mu {\left\|W^{\prime }A\right\|}_{1}$$ (8)

where diag(*W*′) = [*d*_*J*_(*y*,*x*_1_),*d*_*J*_(*y*,*x*_2_),…,*d*_*J*_(*y*,*x*_*m*_)] is a weighted diagonal matrix, and *d*_*J*_(*y*,*x*_*i*_) is the Jaccard distance between *y* and *x*_*i*_.

The weighted matrix *W*′ can be regarded as a locality adaptor of the SR coefficients by using the Jaccard distance between *y* and *x*_*i*_. As a constrained LASSO problem, Eq (8) can be solved by the Least-angle regression (LARS) algorithm, which is described in detail in [21].

To summarize, in the fine recognition stage, we represent the test sample as a linear combination of the candidate training samples *x*_1_,*x*_2_,…,*x*_*m*_ using the new WSRC algorithm Eq (8), where the sparse coefficients *α*_1_,*α*_2_,…,*α*_*m*_ indicate the contribution of the training samples in representing the test sample. Correspondingly the contribution of the *i*^th^ class can be seen as

$$c_{i}=a_{i1}x_{i1}+a_{i2}x_{i2}+\cdots +a_{in_{i}}x_{in_{i}}$$ (9)

where *i* = 1,2,…,*S*, *x*_*ij*_∈*X*_*i*_ is the *j*^th^ training sample of the *i*^th^ class, and *n*_*i*_ is the number of training samples in the *i*^th^ class. The deviation of *c*_*i*_ from *y*, namely the residual of the *i*^th^ class, can be calculated by

$$e_{i}={\left\|y-(a_{i1}x_{i1}+a_{i2}x_{i2}+\cdots +a_{in_{i}}x_{in_{i}})\right\|}^{2}$$ (10)

Clearly, the residual indicates the goodness of fit to the test sample by the weighted sparse representation using the training samples from each candidate class. Thus, *y* can be finely classified into the class that produces the smallest residual.

### Plant species recognition process

**Fig 2:**
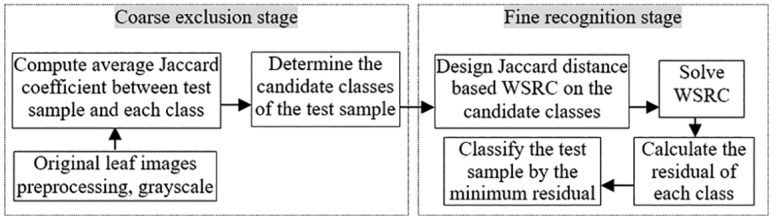
Pipeline of the coarse-fine leaf based plant species recognition method.

To implement our proposed JDSR method, we design the following pipeline as in Fig 2.

The detail steps of the proposed method are summarized as follows:

**Input**: *n* color leaf training images from *C* classes, *q* color leaf test images, the number *S* of candidate classes.

Step 1 Image pre-processing. Convert the leaf images, including both the training and test samples, into grayscale images, and extract their binary orientation images by using the Canny edge detection algorithm [23].

Step 2 For each binary orientation test image *y*, by Eqs (5) and (6), compute its Jaccard coefficient and Jaccard distance with each training image. By Eq (7), calculate the average Jaccard coefficient *J*(*y*,*X*_*i*_) of the *i*th class, *i* = 1,2,…,*C*.

Step 3 Determine *S* classes from the training set with top ranked average Jaccard coefficients as the candidate classes for the test sample recognition. These *S* candidate classes are then used for fine recognition while the rest *C-S* classes are excluded from the training set.

Step 4 Construct the dictionary *G* using the training samples of the *S* candidate classes. The test and training binary orientation images are converted into column vectors for SR calculation.

Step 5 Formulate the weighted *l*_1_-minimization optimization problem as Eq (8) by the weight matrix.

Step 6 Solve Eq (8) and obtain the sparse coefficients.

Step 7 Compute the residual between *y* and its estimation *c*_*i*_ for each class.

Step 8 Classify the test sample into the class that produces the smallest residual.

**Output**: Recognition result. Repeat Steps 2 to 8 for each test sample, calculate the classification rate for each class, and then average the classification rate.

### Discussion

From the above pipeline, the computational complexity of the proposed method mainly depends on two steps: selecting *S* candidate classes and solving the weighted *l*_1_-minimization problem. The time cost in the first step is about *O*(*C* log(*n*)+*n*), and the time cost in the second step is about *O*(*Dm*)+*O*(*Dm*^2^)+*O*(*m*^3^) [10]. Comparing the two time costs, we find that reducing the number of training samples from *n* to *m* in the coarse recognition stage plays a critical role in improving the computational efficiency of the proposed method.

The proposed method is particularly suitable for leaf based plant species recognition on large databases because of the following reasons:

(1) In the coarse recognition stage, a significant number of training samples are excluded from the training set due to their dissimilarity with the test sample, which effectively simplifies the classification problem and reduces the side-effect of outlying training samples. (2) In the fine recognition stage, training samples are weighted by their Jaccard distance to the test sample, which benefits the sparse representation and the classification performance.

## Experiments and result analysis

**Fig 3:**
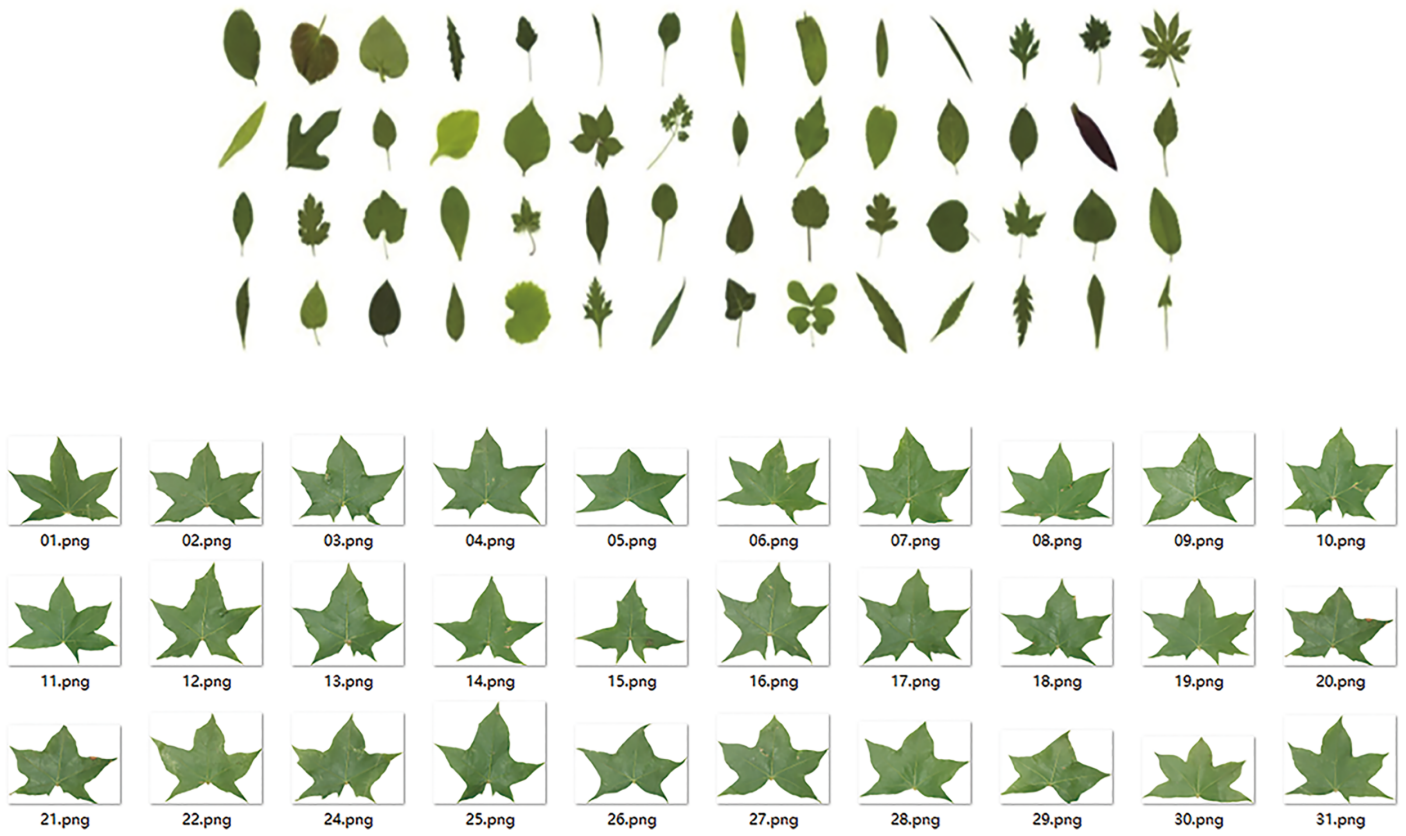
Examples from ICL leaf database. (A) Different kinds of plant leaves, (B) 30 Acer mono maxim leaves from ICL leaf database.

In this section, we conduct a number of experiments using the ICL database, which was constructed by intelligent computing laboratory (ICL) of Chinese Academy of Sciences. The database contains 6,000 leaf images from 200 plant species with 30 leaf images per class, providing a rich resource to verify the effectiveness of the proposed method. Fig 3 shows same examples. For comparison purpose, we also apply the other five plant recognition methods on the same dataset, including three leaf feature extraction based plant recognition methods, i.e., SCTF, which is based on shape, color and orientation feature extraction [5], TSFNC, which uses texture and shape features with neural classifiers [24], and SFCHKNN, which combines shape and color histogram feature extraction with K-nearest neighbor classifiers [25]; two sparse representation based plant recognition methods, i.e., SR using sparse representation of leaf tooth features [11] and LSR standing for learning sparse representation [26]. All experiments are carried out using MATLAB 7.0 software on an Intel Xeon X3430 PC with 2 GB RAM. For SR based methods, the SR coefficients and residuals are calculated using the SRC function in the SR Toolbox (http://sites.google.com/site/sparsereptool).

### Leaf image pre-processing

The original leaf images in ICL database are color images oriented at a random angle and having various shapes, colors and size. In experiments, a lot of preprocessing is performed, such as smoothing, enhancing, denoising, alignment, and scale normalization are required prior to recognition [1,4,5]. Because all leaf images in the ICL database have a simple, low-intensity background, we take a small threshold (30) to separate the background from the leaf image, i.e., the pixel values less than 30 is taken as white under gray level of 255.

Since leaf color changes under varying conditions and does not provide much useful information in automatic recognition, in the following experiments, each original color leaf image is converted into grayscale by,

Gray=0.2989*R+0.5870*G+0.1140*B (11)

where Gray is the grayscale value, R, G and Bare the components of red, green, and blue, respectively.

After converted to grayscale, each image is properly cropped and normalized to the size of 32×16 by singular value decomposition (SVD), enhanced by histogram equilibrium, and then aligned by the shape geometric long axis of the leaf [1,4,5]. These normalization and registration procedures guarantee that the leaf images are in the same space for comparison. Next, for each processed grayscale image, we extract its binary orientation image in the following four steps: (1) convert each colour leaf image into grayscale image, and smooth each grayscale image by the Gaussian convolution; (2) a simple Robert cross operator is applied to calculate the smoothed image difference to highlight the image regions with high first spatial derivative; (3) a dilation operator in morphology operations is used to fill in these holes; (4) a non-maximal suppression process is conducted to track along the top of these ridges and set all pixels off the ridge top to zero.

**Fig 4:**
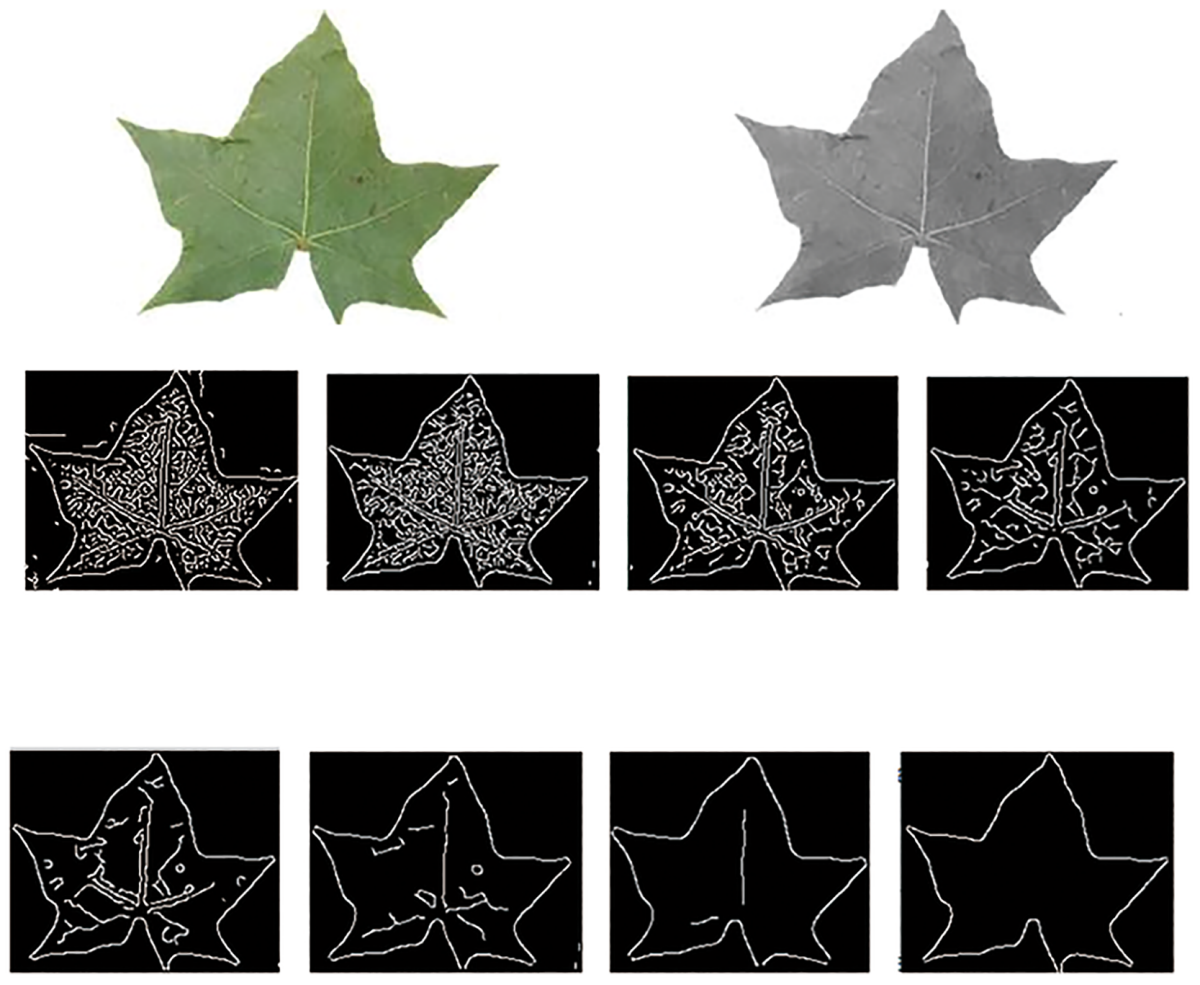
Binary orientation images of an Acer mono leaf under 8 different sensitivity thresholds. (A) Original color image, (B) Grayscale image, (C) Binary orientation images with different sensitivity thresholds *r*.

Fig 4 shows the extracted binary orientation images of an Acer mono leaf by the above pre-processing procedure, using eight different sensitivity thresholds *r*.

Finally, in WSRC, each extracted orientation image is converted into a column vector with the dimensionality *D* = 32×16 = 512.

### Parameter setting

**Fig 5:**
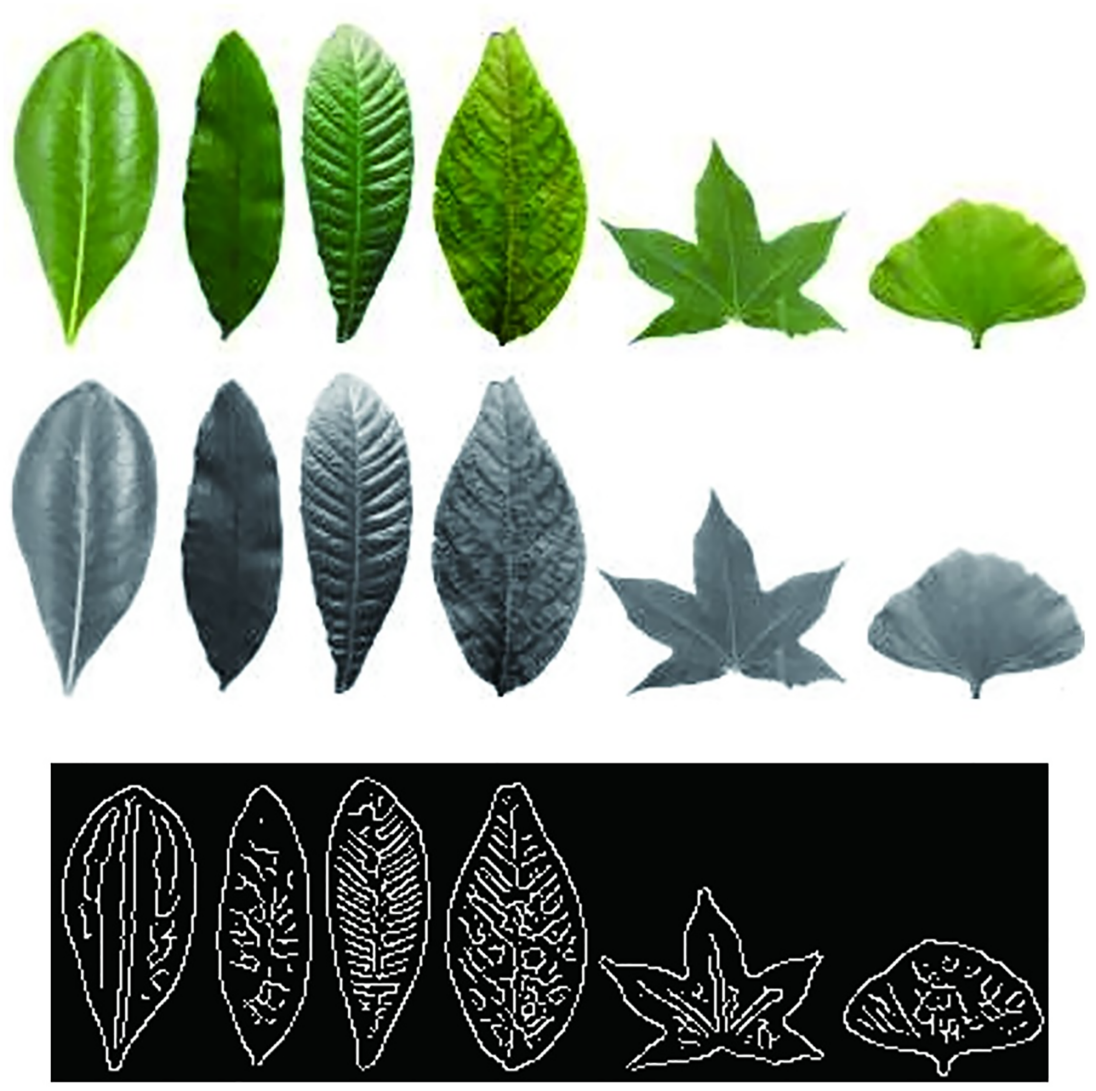
Six leaves from six different species and their grayscale and orientation images. From left to right, the six species are: Pittosporum, Alondra, Loquat, Cherry Blossoms, Acer mono and Ginkgo. (A) Original color images, (B) Grayscale images,(C) Binary orientation images.

In WSRC, when the regularization parameter *μ* = 0, Eq (8) is a standard least squares solution. A larger *μ* leads to increased sparseness of the obtained solutions. Eventually, as *μ*→∞, only a single non-zero coefficient will be admitted in the solution by *A* = *G*^−1^*y*. From Fig 4, it is found that the extracted orientation images vary a lot under different sensitivity thresholds. In order to obtain the optimal threshold for our leaf recognition method, we perform several cross validation experiments. We randomly select 60 samples from 6 different species: Pittosporum, Alondra, Loquat, Cherry Blossoms, Acer mono and Ginkgo, with 10 samples per class, and convert them into grayscale images. Fig 5 shows an example of 6 leaf images from the 6 species. In the function edge (Gray, 'Canny', threshold), we set the threshold to vary from 0.01 to 0.5 with a step size 0.01. Under these total 60 thresholds, the extracted binary orientation images as shown in Fig 5C. Each threshold, the leave-one-out cross validation is conducted by implementing WSRC based leaf recognition experimentand dictionary constructed by 59 training samples.

**Fig 6:**
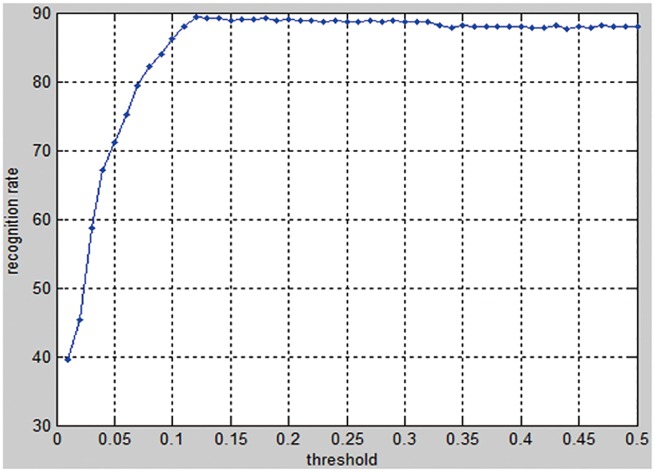
The recognition rates versus the thresholds.

Fig 6 shows the classification rates under different thresholds. From Fig 6, it is found that the proposed method achieves the highest recognition rate when the threshold is 0.12, where *μ* = 0.001 [11,20]. This inspired us to set the threshold to 0.12 in the function edge (Gray, 'Canny', threshold) in the following experiments.

To observe the effect of *μ* on the recognition rate, we select *μ* as 0, 0.00001, 0.0001, 0.001, 0.01, 0.1 and 1 in SRC, and the results are shown in Table 2. From Table 2, we find when *μ* is zero, the recognition rate is very low, because at this time, SRC ignores the sparse constraint condition. Because of the similarity of plant leaves, the recognition rate is poor. When *μ* is less than 1, the recognition rate changes slightly, and when *μ* is relatively large, the sparse dominant the optimal problem, then the recognition rate reduces a little, and when *μ* is 1, the recognition rate is reduced to 94.21%. In the following experiments, the default value of *μ* is 0.001 in the following experiments.

**Table 2:** The recognition rates change versus *μ*.

*μ*	0	0.00001	0.0001	0.001	0.01	0.1	1
Recognition results	46.31	94.51	94.32	94.37	94.48	94.27	94.21

### Experiments

After the above image pre-processing, all 6,000 leaf images from 200 species in the ICL database are converted into binary orientation vectors. These vectors are divided into training sample set and test sample set based on five-fold cross validation. The training set is used to train the plant recognition model, while the test set is used to validate the performance of the proposed method. In the cross validation, the 30 binary vectors from each species are randomly partitioned into 5 equal sized subsets, of which 4 subsets are used as the training set and the remaining one are used as the testing set.

For each test sample, we calculate its Jaccard coefficient with each training sample by Eq (5), and obtain the average Jaccard coefficient with each training class by Eq (7). We then select *S* classes from the training set with the largest average Jaccard coefficients. In practice, *S* is set to be half of the number of all training classes, i.e., *S* = 200/2 = 100. That is to say, 100 classes are excluded, thus100 classes are selected as the candidate classes with 24 training samples per class, i.e., there are 2400 candidate training samples to construct the over-complete dictionary for SRC.

By the Jaccard coefficient between the test sample and each training sample, the Jaccard distance is easily calculated by Eq (6), which is then used to formulate the weighted mixed *l*_2_−*l*_1_-norm minimization problem as Eq (8). Solving the constrained LASSO problem [17], we obtain the sparse coefficients and calculate the residuals for each candidate class. Finally, the test sample is classified into the class with the smallest residual.

We conduct 10 independent five-fold-cross validation experiments, and obtain 5×10 = 50 experiment results. Table 2 presents the average of the 50 experiment results. For comparison, Table 2 also lists the recognition results of the other 5 methods.

### Result analysis

From Table 3, it is found that the proposed method outperforms the three leaf feature extraction based plant recognition methods and two SR based plant recognition algorithms, and achieves the highest average recognition result and shortest running time. The possible explanation is that, all the five comparison methods essentially rely on extracting and selecting image features for plant recognition. However, it can be seen from Fig 3 that the 30 Acer mono leaves are very different from each other, so it is difficult to extract useful features from each leaf image that are representative for the species. Moreover, the feature extraction process is time consuming. In contrast, using the proposed method, half of the training samples are quickly excluded by the Jaccard coefficient in the coarse recognition stage, which can largely reduce the computational cost and eliminate the side-effect of outlying samples on the classification decision, and in the fine recognition stage, using the Jaccard distance (i.e., dissimilarity) to linearly represent the test sample leads to a sparser representation than other classical SR algorithms. In addition, compared with SR and LSR, the proposed method represents the *l*_1_-minimization optimal problem with relatively smaller number of candidate training samples, thereby saving much computational time.

**Table 3:** Average recognition rate, standard deviation and running time of SCTF, TSF, SFCHKNN, SR, LSR and the proposed method.

Method	SCTF	TSFNC	SFCHKNN	SR	LSR	Our method
Recognition results	84.63±3.42	87.52±3.14	86.26±2.86	89.15±3.25	90.28±2.76	93.42±2.54
Running time(*s*)	108	114	98	115	121	79

## Conclusions and future works

Leaf based plant recognition is an important and challenging topic. In this paper, a novel coarse-to-fine plant species recognition method is proposed using Jaccard coefficients and Jaccard distance based WSRC. The proposed method includes two stages. Firstly, the average Jaccard coefficient is used to determine candidate classes that are relatively ‘close’ to the test sample. Secondly, the test sample is finely classified by Jaccard distance based WSRC. Experiment results on the ICL dataset show that the proposed method achieves improved classification rate and reduced computational time in plant recognition. It is worth noting that, since the proposed method is based on the binary orientation images rather than some transformation-invariant features, the image registration step is required prior to the calculation of Jaccard coefficient and distance. Otherwise, the recognition result would be unreliable. In our current practice, each leaf image is aligned by the shape geometric long axis. Though resulting in good recognition performance in our experiments, this alignment may not be efficient for a large number of leaf images. Learning a dictionary directly from the training data usually leads to better representation and can provide improved results in many practical image processing applications such as restoration and classification. Our future work will be focused on dictionary learning and learning a dictionary directly from the training data rather than using a predetermined dictionary.
